# Comparing root exudate collection techniques: An improved hybrid method

**DOI:** 10.1016/j.soilbio.2021.108391

**Published:** 2021-10

**Authors:** Alex Williams, Holly Langridge, Angela L. Straathof, Graeme Fox, Howbeer Muhammadali, Katherine A. Hollywood, Yun Xu, Royston Goodacre, Franciska T. de Vries

**Affiliations:** a School of Earth and Environmental Sciences, The University of Manchester, Oxford Road, M13 9PT, Manchester, UK; b Ontario Soil and Crop Improvement Association, 1 Stone Road West, N1G 4Y2, Guelph, Ontario, Canada; c Ecology and Environment Research Centre, Department of Natural Sciences, Manchester Metropolitan University, Manchester, UK; d Department of Biochemistry and Systems Biology, Institute of Systems, Molecular and Integrative Biology (ISMIB), University of Liverpool, Biosciences Building, Crown Street, L69 7ZB, Liverpool, UK; e Manchester Institute of Biotechnology, The University of Manchester, Princess Road, Manchester, M1 7DN, UK; f Institute of Biodiversity and Ecosystem Dynamics, University of Amsterdam, PO Box 94240, 1090 GE, Amsterdam, the Netherlands

**Keywords:** Root exudates, Root traits, Rhizosphere, Plant-microbe communication, Carbon

## Abstract

1. Plant-microbe interactions are critical for ecosystem functioning and drive rhizosphere processes. Root exudates are an important soil carbon (C) input, as well as a mechanism for communication between plants and rhizosphere microbes, but are notoriously difficult to extract and characterise. Common methods produce either substantial noise from the soil or do not mimic natural systems. Optimising methods for root exudate collection in soil is crucial for advancing our understanding of root-microbe interactions under changing environmental conditions.

2. Hybrid root exudate collection methods, where plants are grown in soil and transferred to hydroponics for exudate collection after root washing, might offer an ecologically relevant alternative to existing approaches. However, this method causes potential root damage as well as osmosis and subsequent leaking of cell contents. Here, we assessed different ‘root recovery’ periods after root washing and before hybrid root exudate collection, by comparing root exudate quantity and quality with both damaged root extracts and with leachates collected from the intact root-soil system. This was done across three common grassland species representing three functional groups.

3. We found that root exudate profiles of the shortest recovery period (0 days) were similar to damaged root extracts and were very high in C. With an increasing period of root recovery, profiles were more similar to leachates collected from the intact root-soil system, and C concentrations decreased. While both hybrid and leachate collection methods separated species by their root exudate profiles, the hybrid method was less variable in terms of the amount of C measured and provided a more diverse and abundant metabolome with better identification of metabolites.

4. Our results show that a recovery period after root washing of at least 3 days is critical to prevent root damage bias in hybrid collection methods, and that our hybrid method yields exudates that discriminate between species. Our data also suggest that exudates collected with this hybrid method are ecologically valid, which is vital for gaining a mechanistic understanding of their role in ecosystem functioning.

## Highlights

• Root-washing method is suitable for ecologically sound root exudate collection.
• It allows accurate measures of exudate quantity and composition.
• Damage affects must be accounted for and use of a recovery protocol is recommended.
• The method permits identification of metabolite specificity between species.
• Leachate complements, provides rhizosphere chemical info and species specificity.

## Teaser

Tweetable abstract: We introduce a root-washing method for ecologically sound root exudate collection to minimise root damage and distinguish species.

## 1 Introduction

The rhizosphere is a diverse and heterogeneous bio-chemical network, where roots and micro-organisms cohabit in a complex interactive community. Much of the carbon (C) input into the rhizosphere and surrounding soil derives from root exudation. This is an active process whereby complex cocktails of phyto-metabolites are released by plants to serve both well established and enigmatic roles in plant-microbe communication (Canarini et al., 2019). Understanding root exudation is essential to shed light on rhizosphere communication and promote a better understanding of plant-soil interactions, especially in response to increasingly common disturbances. Root exudates shape microbial community composition and activity in the rhizosphere, which impacts short and long-term adaptation to disturbances such as drought (Williams and de Vries, 2020; Xu and Coleman-Derr, 2019) and pathogen challenge (Doornbos et al., 2012). The association of plants with these microbes is also functionally important for determining soil carbon (C) and nitrogen (N) cycling processes through enhancing soil organic matter decomposition, altered nitrogen cycling, and phosphorus mobilisation in the rhizosphere (Coskun et al., 2017; Finzi et al., 2015; Richardson et al., 2009). Hence, improving our understanding of exudation processes is key to evaluating future functioning of our agricultural and natural ecosystems.

The functional control that plants exhibit over rhizosphere communities and processes is driven by the composition of root-exuded metabolites that microbes depend upon. Identifying the chemical constituents of root exudates is central to identifying plant-microbial interactions that maintain plant health and survival. For example, benzoxazinoids increase maize root colonisation of the plant growth promoting rhizobacteria *Pseudomonas putida* (Neal et al., 2012), and certain phenolic compounds have been illustrated to prime forest soils for organic matter decomposition by stimulating shifts in bacterial community composition (Zwetsloot et al., 2020). Additionally, malic acid is chemotactic for systemic-resistance inducing *Bacillus subtilis* (Rudrappa et al., 2008) and glycerol-3-phosphate stimulates root-colonisation of monoderm bacteria to enhance resistance during drought (Xu et al., 2018). Equally, root exudation is a dynamic process and may select for specific microbial communities at specific growth stages (via exuded aromatic compounds; Zhalnina et al., 2018). Together these interactions not only have implications on soil ecosystem structure and resilience (Png et al., 2019) but also plant soil feedback and long-term ecosystem maintenance (Teste et al., 2017; Williams and de Vries, 2020) as well as soil function and stability (including nitrification ability - Guyonnet et al., 2017 - and stress resilience - Vries et al., 2020). However, for many processes under the influence of root exudates, the acting compounds have not yet been identified. In part this is because the sampling of root exudates is challenging; small molecules tend to be found at low concentrations and may be absorbed onto soil components or rapidly consumed by rhizospheric microbes, which affects their recovery and analysis.

Typical methods to collect root exudates employ either artificial (hydroponics), semi-natural (leachate) collection techniques on whole-root systems, or in-field methods that either extract a root section from its soil matrix (*e.g.* Phillips et al., 2008; Michalet et al., 2013), or plant saplings in rhizotrons (*e.g*. Zang et al., 2014) for *in situ* exudate collection. Distinguishing specific metabolites in a soil matrix is difficult: access to roots is impeded by the soil matrix and exuded compounds are unlikely to persist for long in conditions where they can be metabolised by microorganisms (Kuijken et al., 2015). Indeed, soil microorganisms use, breakdown and rapidly restructure plant-derived metabolites into a vast signalling network of secondary metabolites (Mommer et al., 2016; Sasse et al., 2018). Furthermore, rhizosphere processes driven by root exudation may stimulate the release of dissolved organic carbon (DOC) that originates from soil organic matter, further confusing downstream analysis and interpretation (He et al., 2006). As a result, most researchers use hydroponic collection methods, which provide a repeatable, high confidence and specific identification of metabolites present in root exudates (Strehmel et al., 2014; van Dam and Bouwmeester, 2016). *Arabidopsis thaliana* exudates, for instance, contain a diverse range of compounds in hydroponics (Narasimhan et al., 2003; Strehmel et al., 2014) – including communication molecules such as coumarins, and defence related compounds such as glucosinolates, salicylic-acid and jasmonic-acid. However, growth in hydroponics profoundly affects root morphology (Ascough and Fennell, 2004), physiology (Sgherri et al., 2010) and, critically, the whole organism response to environmental stimuli (for instance toxicity tolerance, Tavakkoli et al., 2010; Chen et al., 2018), resulting in data with limited application in natural systems.

In addition to being affected by host genotype (Mönchgesang et al., 2016) and plant growth stage (Aulakh et al., 2001), root exudation chemistry is likely determined by the soil environment (Canarini et al., 2016; Iannucci et al., 2017; Williams and de Vries, 2020), including soil microbial community composition and activity (Aulakh et al., 2001; Chaparro et al., 2013). Thus, properly elucidating the functional role of root-exuded metabolites requires study in natural soil environments. It has previously been illustrated that many phytogenic secondary metabolites involved in microbial communication, such as phenyl propanoids and benzoxazinoides, were present in the leachate of intact root-soil systems of *A. thaliana* and *Zea mays* (Pétriacq et al., 2017). Although certain classes of secondary metabolites measured were also present in *Arabidopisis* exudates from hydroponic cultures (Narasimhan et al., 2003), many were not, suggesting that the absence of a natural soil matrix limits comprehensive profiling of root exudation chemistry. However, the extent to which specific metabolites are plant derived *versus* those derived through microbial metabolism/degradation, or through edaphic processes such as DOC release, remains unclear. Hybrid root exudate collection methods, where plants are grown in soil before their root systems are washed and transferred to a hydroponic solution for exudate collection, might help address this issue (*e.g.* Canarini et al., 2016; Oburger and Jones, 2018).

Hybrid collection methods permit an ecologically relevant assessment of root exudates because roots grow and develop in soil but microbial breakdown of metabolites and interference of soil DOC signals are constrained in hydroponic collection solution. Hybrid methods have been used to characterise changes in exudate composition over the life-cycle of rice (Aulakh et al., 2001) and lupine (Lucas García et al., 2001), as well as the composition role of exudates in determining plant species pH tolerance (Ström et al., 1994). In addition, hybrid methods allow the application of treatments that cannot be applied to a hydroponic system, such as drought - which was shown to induce changes in exudation rate in sunflower (*Helianthus annus*) and exudate composition in soybean (*Glycine max*; Canarini et al., 2016). Furthermore, exudates collected with a hybrid method have been reapplied to soil to quantify their impact on soil-microbe respiration (de Vries et al., 2019), which underscores the method's potential for unravelling the mechanistic role that certain root-exuded compounds play in the rhizosphere. However, despite these advantages, hybrid methods introduce bias as root washing causes unavoidable stress and root damage (Oburger and Jones, 2018) - although the extent to which this impacts exudate composition and rate is not clear. Many studies collect exudates immediately after cleaning, without any recovery period (excluding the single root exudation trap method which does; Phillips et al., 2008) and do not account for the impact of potential damage on the composition of root exudates.

Here, we compare hybrid exudate collection methods with leachates of the intact soil-root system (which allow for a holistic characterisation of rhizosphere chemistry). We also evaluate the efficacy of the hybrid collection method by comparing different root recovery durations after washing (no recovery and short- and longer-term recovery) to extracts of roots that have been physically damaged. We hypothesise that without a period of recovery, root exudates from the hybrid collection method will be confounded by damage related signals induced by root washing. Hence, we hypothesise that with increasing recovery time the similarities between hybrid collection methods and damaged root extracts will decrease, both in terms of quantity (C content) and quality (metabolic profile) of the root exudates. In addition, because exudates collected with hybrid methods have limited microbial degradation, and do not contain complex edaphic signatures, we hypothesise further that the hybrid method will yield more appropriate, and informative, species-distinct chemical profiles than with leachate. We test these hypotheses in a controlled factorial experiment with three common grassland species.

## 2 Methods

### 2.1 Experimental design

We collected top-soil, a brown earth over limestone bed-rock (clayey brown earth soil from the Wilcocks 1 association; means ± standard deviation of %N 0.57 ± 0.02, %C 5.70 ± 0.14%, pH 5.35 ± 0.3), from mesotrophic grassland with a management history of light grazing and minimal fertiliser input at Colt Park in northern England (54°11′37.1″N 2°20′54.9″W, 348 m above sea level) in March 2016. After collection, soil was sieved and homogenized (4 mm mesh size) and stored at 4 °C until further use.

We chose three common European grassland species that represent the functional groups grass, forbs and legumes: Yorkshire fog (*Holcus lanatus* L.), common sorrel (*Rumex acetosa* L.) and small-leaved white clover (*Trifolium repens* L.), respectively. All seeds were sourced from Emorsgate Seeds (Norfolk, UK). Seeds were stratified at 4 °C before being seeded into plug trays kept in the University of Manchester Firs botanical grounds greenhouse facility (Manchester, UK). After two weeks, individual seedlings (*n* = 25 per species) were transplanted into 500 mL experimental pots (105 mm diameter, 75 mm depth) with 400 g of field-moist sieved and homogenized soil (equals ~160 g dry soil), and kept in a greenhouse for a further three months (from February to May 2017 with prevailing spring-time growth conditions) in a randomized 5-block design (blocks contained 5 individuals of each species, totalling 15 pots per block). Pots were watered by weight to maintain a constant soil moisture of approximately 60% of water-holding capacity throughout the experiment.

After three months, all plants were transferred to Percival AR-66L2 climate chambers (CLF PlantClimatics, Wertingen, Germany) set at long day (16 : 8 h, light: dark at 16 °C night and 18 °C day; air relative humidity 65%) and left for two weeks to standardise growth conditions for each species before roots were repeatedly washed by careful suspension and shaking in water, with sediment and organic debris being tweezed out, until all adhering material was removed. After root washing plants were subjected to one of five treatments (*n* = 5 per treatment): 1) roots were damaged by multiple crushing motions of pestle and mortar for 5 min, until roots were pulpy, before exudate collection (D); 2) root exudates were collected immediately after root washing (H0); 3) roots were washed and plants were transferred into an aerated hydroponics solution back to the same climate chamber and conditions to let roots recover from washing for 3 days before root exudate collection (H3); 4) roots were washed and plants were transferred into an aerated hydroponics solution to let roots recover from washing for 7 days before root exudate collection (H7); 5) leachates were collected from the intact root-soil system (L). Hydroponics solution for recovery was a made from a slurry of 200 g of stored field soil in 1 L of MilliQ water that was left to settle and filtered (0.2 mm mesh).

### 2.2 Root exudate and leachate collection

For hydroponic collection (including D and H0) plants were suspended in sterile glass jars with their roots submerged in 100 mL of collection solution (pure milliQ water) on ice (to minimise turnover of collected exudates) and agitated at 60 rpm on a Stuart Orbital shaker (Cole-Parmer, St. Neots, UK) for 2 h in natural ambient light at 18 °C; thereafter the exudate solution was syringe filtered at 0.22 μm (Merck Millipore). Leachates were collected by slowly pouring MilliQ over the soil surface of the intact plant-soil system until 100 mL of leachate (L) was collected, on ice, from under the pot. This leachate was syringe filtered at 0.22 μm (Merck Millipore). For every collection method 90 mL of each sample was split into three 50 mL CellStar Falcon tubes (227261, Greiner Bio-one, Gloucester, UK). All samples were promptly frozen to −80 °C then freeze-dried in a Scanvac CoolSafe 55-9 Pro freeze-drier (LaboGene, Lynge, Denmark) and returned to the −80 °C for storage. To double the concentration of exudates pellets of two freeze-dried tubes per sample, were resuspended in 10 mL LCMS-grade water (CHROMASOLV, Honeywell, Bucharest, Romania) and combined before being freeze dried again. The third tube was resuspended in 20 mL ultra-pure water for determination of total carbon using a TOC-L analyser (Shimadzu, Kyoto, Japan) with the combustion catalytic oxidation method.

### 2.3 Root trait analyses

After exudate, or leachate, collection intact and complete root systems were quickly rinsed with water before separation from the aboveground tissue, which was weighed for fresh biomass and, after 48 h at 60 °C, dry biomass. Roots, still joined at the root crown, were carefully teased apart and evenly spread out in deionised water on a transparent Perspex tray (300 × 200 mm). Root systems were then imaged at a resolution of 600 dpi with an Epson Expression 11000XL flatbed scanner system. Structural root traits (total root length, average root diameter and root volume) were analysed using the 2013 WinRHIZO® pro software (Régent Instruments Inc., QC, Canada) using the batch analysis feature for each species. A correction was applied to account for roots crossing and debris smaller than length/width ratio of 4 were excluded from the analysis. These settings were manually verified and the level of variation between methods was consistently below 5%.

After analysis, roots were blotted dry, weighed, and dried at 60 °C for 48 h before re-weighing for dry matter content. These measurements were used for calculating of specific root length (SRL; root length per unit dry root mass mm g^−1^), root tissue density (RTD; dry root mass per unit root volume g cm^−3^), root surface area (SA) and root dry matter content (RDMC; the ratio of dry root mass: fresh root mass mg g^−1^). Representative subsamples of the dry root system, as well as aboveground tissue, were ground using a MM400 ball mill (Retsch, Hope Valley, UK) after which 5 mg were weighed using an XP6 micro-balance (Mettler Toledo, Leicester, UK) to be analysed for tissue C and N content using a Vario Micro Cube (Elementar, Germany).

### 2.4 Mass spectrometry analysis using gas chromatography (GC-MS)

#### 2.4.1 Derivatisation and run

The remaining two freeze-dried exudate pellets were resuspended in 1 mL of LC-MS grade water and transferred into new 2.5 mL microcentrifuge tubes. At this point, equal aliquots (5 μL) from all samples were combined in a new tube, per each sample type (leachates, and hydroponics), to be used as quality control (QC) sample, followed by addition of 100 μL of internal standard (0.2 mg mL^−1^ of succinic-d_4_ acid, and glycine-d_5_) to all samples before being lyophilised overnight using a speed vacuum concentrator (Concentrator 5301, Eppendorf, Cambridge, UK). All dried extracts were derivatized by oximation followed by a silylation step, using methoxyamine-hydrochloride in pyridine and N-Methyl-N-(trimethylsilyl) trifluoroacetamide, respectively. Metabolomics data were acquired using a 7890 B GC coupled to a 5975 series MSD quadrupole mass spectrometer, equipped with a 7693 auto-sampler and piloted by Chemstation software (Agilent, Technologies, UK).

#### 2.4.2 Data pre-processing

Raw output folders obtained from Chemstation (Agilent MassHunter) were converted to mzXML format using MSConvert software, with peak picking enabled with the Vendor algorithm. These mzXML files were then deconvolved and aligned using the eRah package in R. A missing compound recovery step was taken to ensure a complete representation of all metabolites that were present in at least 3 samples. Four datasets were obtained this way for down-stream analysis; one containing only leachate samples, one containing damage and hybrid collection samples, one combined dataset with all samples and a final dataset only containing data from the hybrid collection samples. These datasets consisted of 135, 761, 547 and 687 unique metabolite features respectively. The combined dataset including leachates and hydroponics was used to visualise global differences between treatments using PCA. As the classes of metabolites identified were very different, leachate and hydroponics data were subsequently analysed separately. To correct for drift, batch and GCMS injection order for each dataset, QC correction was implemented according the procedure described in Dunn et al. (2011) using an inhouse script for MATLAB (Mathwork, MA; https://github.com/Biospec/cluster-toolbox-v2.0). This correction was not performed on the final dataset as QCs were biased from damaged samples and were inappropriate for these data. Finally, to account for variation of root biomass between species, metabolomics relative abundance data were standardised per mass of root dry weight.

#### 2.4.3 Statistical analyses

GC-MS and C content data were log_10_ transformed to meet the requirements of statistical models and to normalise distribution across putative metabolites. To test for differences between collection methods and between species (along with the interaction), we ran ANOVA models on above-ground and below-ground biomass data, root and foliar CN content data, root and leaf water content and C content of the exudates. All models were run with species and collection method as main factors, with an error term fitted for block, followed by Tukey HSD tests for significant differences between groups and with a significance value > 0.05. Principal component analysis (PCA) was performed to visualise treatment effects on root traits. Loadings are presented with arrows to show which root-traits are responsible for the greatest separation between plant species. PCA was applied to standardised GC-MS data followed by PERMANOVA (adonis function in vegan package; Oksanen et al., 2011) with species and collection method as main factors, as well as their interactions (permutations set at 999) to assess their impact on root traits and root exudate profiles, quantified by GC-MS. It should be noted that pseudo-R^2^ (_p_*R*^*2*^) values are being reported with PERMANOVA, which are not comparable to common R^2^.

To visualise the effect of species and root exudate collection method on standardised exudate metabolite profiles, we generated cluster heatmaps, based on *K*means clustering of mean metabolite intensity for each species (pheatmap package; Kolde, 2012). A dendrogram was also applied to this heatmap to illustrate the relative clustering between collection methods. To further quantify the extent to which metabolites differed between D and hybrid recovery methods, univariate analyses (t-tests) were performed on log^10^ transformed data for every metabolite comparing D against H0, H3 and H7. P-values were corrected for multiple comparisons using Benjamini-Hochberg false discovery rate (FDR) correction. Volcano plots were employed to obtain putative identities from all three binary comparisons (D *vs* H0, D *vs* H3 and D *vs* H7) by selecting significant metabolites (-log_10_ *p*-value > 5) that also had a log_2_ fold-change greater than 0.5 or below −0.5). These analyses were performed using the R package MetaboAnalystR (v. 3.0). To measure the extent to which the identities of these metabolites overlapped between each binary comparison, they were assessed for overlap using Venn diagrams.

In addition, to obtain the metabolites that provide the greatest source of discrimination between species we performed supervised sparse partial least-square discriminant analyses (sPLS-DAs) using the mixomics package (Rohart et al., 2017). These analyses were performed in H0, H3 and H7 separately to obtain identities of the metabolites most responsible for species separation in each of these methods. sPLS-DA models were used, although cross-validation could not be accurately performed on the limited number of samples we used in this study – which may be a shortcoming of the method. In each case two components were analysed and the top 20 that best explain the variation on each axis were annotated. Mean values for each metabolite were then coloured by relative intensity between species and presented in heatmaps. Identification of all metabolites was performed using the GOLM database, where we only retained annotations of >80% match factor, so all identification are putative which is level 2 of the Metabolomics Standards Initiative (Sumner et al., 2007). All analyses were done in R (R Core Team, 2013).

## 3 Results

### 3.1 Plant traits

**Fig. 1:**
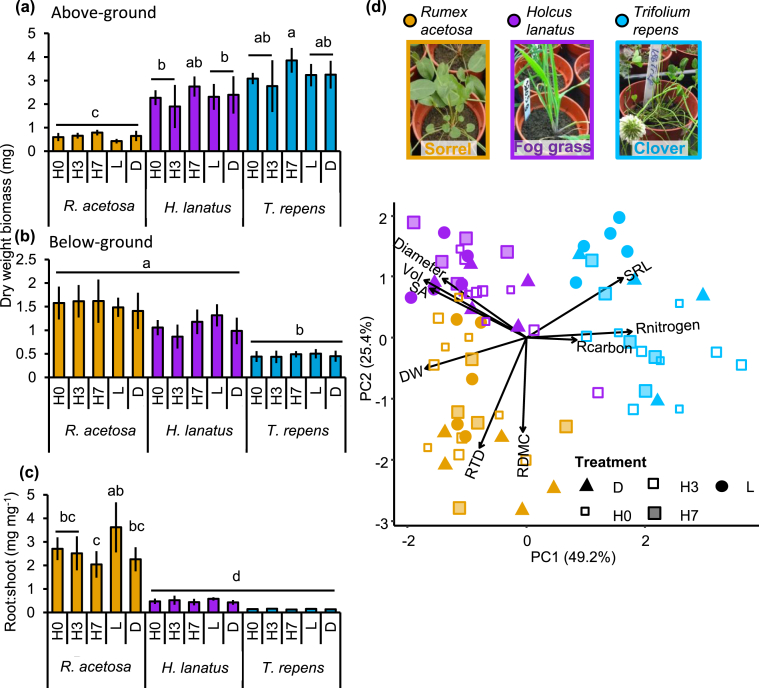
**Biomass and root traits of *R. acestosa*, *H. lanatus* and *T. repens* across exudate collection methods.** Aboveground biomass (a), belowground biomass (b), and the ratio between the two (root:shoot; c) across the five collection methods; PCA scores plot of root traits (d). Bars indicate mean ± standard deviation (*n* = 5 samples) and different letters indicate significant treatment differences. Arrows on PCA indicate projections for each individual trait, and values in parentheses on the axes are total explained variance. Abbreviations: H0, exudates collected immediately after root-washing; H3, hydroponics with 3 days recovery; H7, hydroponics with 7 days recovery; L, leachate collection; D, damaged followed by immediate collection. Vol, root volume; SA, root surface area; DW, root dry weight; RTD, root tissue density; RDMC, root dry matter content; SRL, specific root length.

Collection method mildly affected above-ground biomass (ANOVA for main effect of collection method, *F*_4, 59_ = 3.2, *p* = 0.018; Fig. 1a), which was higher in H7 than in the other collection methods in both *H. lanatus* and *T. repens*, but not *R. acetosa*. This effect was not reflected in below-ground biomass (Fig. 1b) or root:shoot ratio (Fig. 1c). However, in *R. acetosa* root:shoot ratio was highest in L and significantly lower in H7 (ANOVA for main effect of collection method, *F*_4, 59_ = 4.3, *p* = 0.004). Overall, biomass clearly differed between plant species (ANOVA, main effect of species. *F*_2, 59_ = 145.7, *p* = 2 × 10 ^−16^), with *R. acetosa* having lower aboveground biomass than *H. lanatus* and *T. repens*, and *T. repens* having lower below-ground biomass and a lower root:shoot ratio than *R. acetosa* (ANOVA for main effect of species, *F*_2, 59_ = 116.8, 2 × 10 ^−16^ and *F*_2, 59_ = 261.0, 2 × 10 ^−16^, respectively). *H. lanatus* also had a lower root:shoot ratio than *R. acestosa*, but below-ground biomass did not differ. Interestingly, *T. repens* had a lower total water content than the other species (Supp. Fig. 1 a-c).

The collection method seemed to have a significant, albeit weak, effect on root trait expression (PERMANOVA for main effect of collection method, *F*_4, 58_ = 2.8, _p_*R*^2^ = 0.04, *p* < 0.014, Fig. 1d), but species strongly differed in their root trait syndromes (PERMANOVA for main effect of species, *F*_2, 58_ = 91.7, _p_*R*^2^ = 0.68, *p* < 0.001), with a significant interaction between (PERMANOVA for species x collection method, *F*_8, 58_ = 2.0, _p_*R*^2^ = 0.06, *p* = 0.019). Most of the variation in root traits was explained by two principal components (Fig. 1d, PC1 = 50.6% and PC2 = 35%), with the third explaining much less (PC3 = 7%). Separation between species was driven by higher RDMC and RTD in *R. acetosa*, higher diameter, SA and root length in *H. lanatus* as well as high SRL and root C and N, in *T. repens*. In addition, leaf C:N ratio showed a significant effect of species (linear model *F*_2,68_ = 104.52, *p* < 0.001) and was much lower in *T. repens* than in the other two species (Supp. Fig. 1d; raw root trait data are included in Supp Fig. 2).

**Fig. 2:**
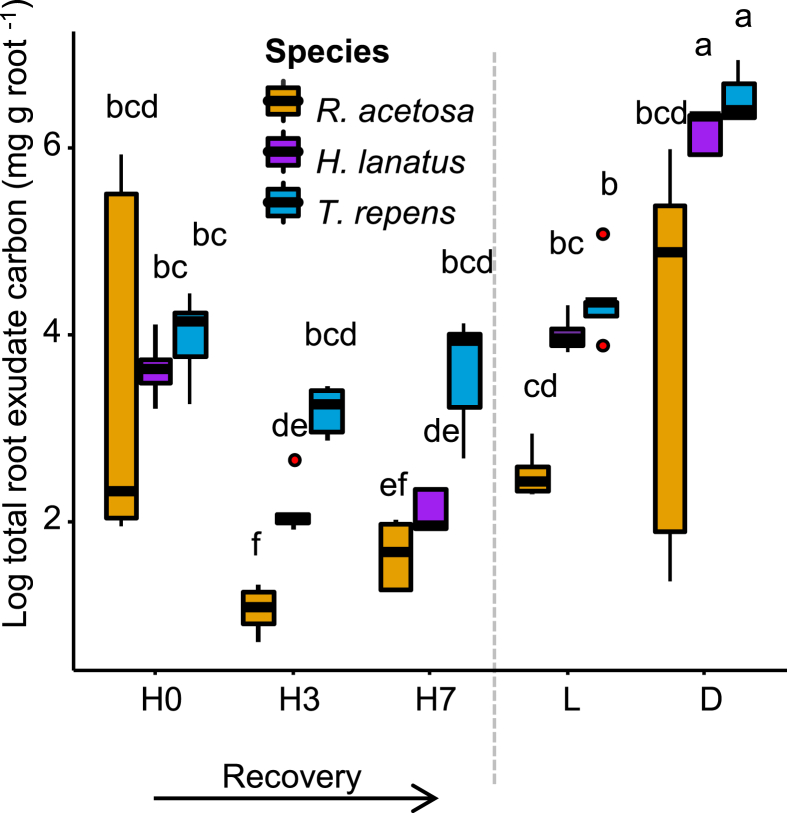
**Total carbon in collected root exudates standardised by root weight for the three species and different collection methods.** Boxes indicates 25–75th percentile interquartile range (IQ), with the median indicated by the black bar and 1–3 x IQ indicated by bars, red dots are outliers (*n* = 5 samples). Different letters indicate significant differences. Abbreviations: H0, exudates collected immediately after root-washing; H3, hydroponics with 3 days recovery; H7, hydroponics with 7 days recovery; L, leachate collection; D, damaged followed by immediate collection. Grey dotted line is to visually separate the recovery treatments (H0 – H7) from the L and D treatments. (For interpretation of the references to colour in this figure legend, the reader is referred to the Web version of this article.)

### 3.2 Leachate and exudate C content

There were stark effects of root exudate collection method on the amount of C collected, with exudates from damaged roots (D) having by far the largest quantities of C (averages of 35.8, 24.9 and 7.7 mg g dry root^−1^ hour^−1^ for *T. repens*, *H. lanatus* and *R. acetosa* respectively; ANOVA main effect of collection method, *F*_4, 59_ = 64.5, *p* = 2 × 10^−16^; Fig. 2, Supp. Table 1). Leachates and H0 also had high C concentrations (between 4.4, 2.8 and 0.6 mg g dry root^−1^ hour^−1^ for leachates and between 2.9, 2 and 6.6 mg g dry root^−1^ hour^−1^ for H0), while concentrations decreased with increasing recovery time after washing (1.3, 0.4 and 0.1 mg mg g dry root^−1^ hour^−1^ for H3 and 2.1, 0.4 and 0.3 for H7; Fig. 2). The three species differed strongly in the amount of C exuded per unit root biomass (ANOVA main effect of species, *F*_2, 59_ = 11.9, *p* = 4.9 × 10^−5^; Fig. 2). Across exudate collection methods, *T. repens* consistently exuded the highest amount of C per g root and *R. acetosa* the lowest. Interestingly, exudation rate of *R. acetosa* was not affected by collection method (likely due to high variability in D and H0), whereas the other two species showed significantly lower C concentrations in all other treatments compared to D.

**Table 1:**
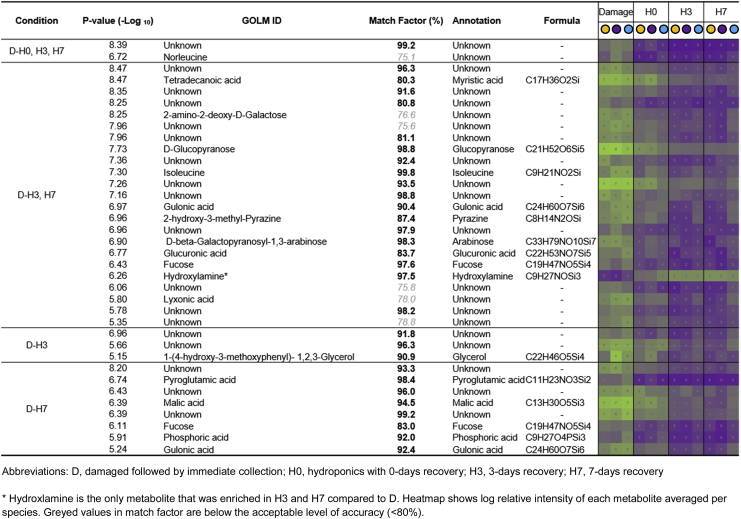
**Identities and level 2 annotations of metabolites significantly enriched in damaged plants.** Numbers within heatmap indicate log-values of metabolite intensity.

### 3.3 Leachate and exudate metabolic profiles

**Fig. 3:**
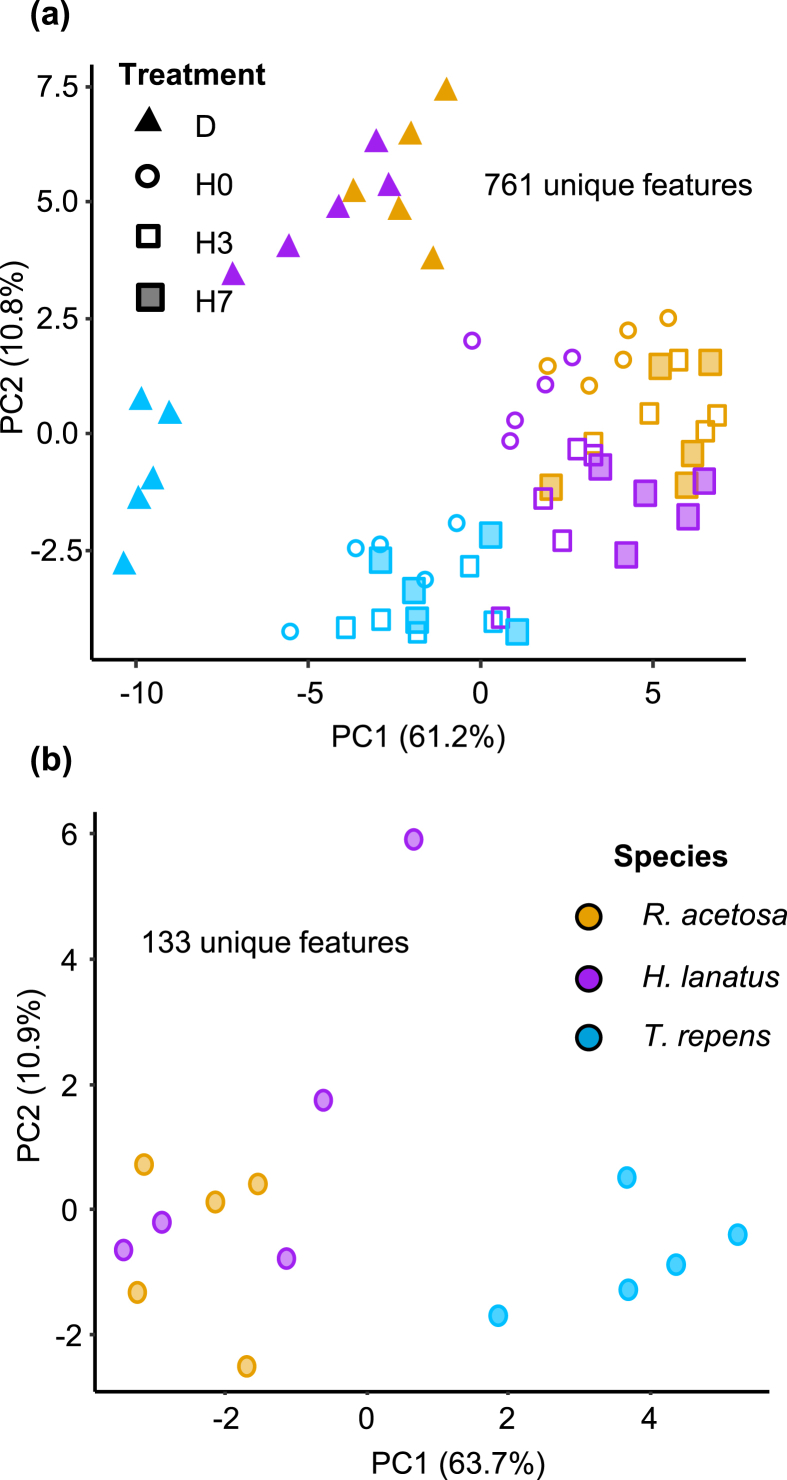
**PCA score plots of root exudate profiles across different species and collection methods**. Comparison between hybrid collection methods and damaged plants (a) and leachate (b). Colours indicate different species and shapes indicate different collection methods. Values in parentheses on the axes are total explained variance. Abbreviations: H0, exudates collected immediately after root-washing; H3, hydroponics with 3 days recovery; H7, hydroponics with 7 days recovery; L, leachate collection; D, damaged followed by immediate collection. (For interpretation of the references to colour in this figure legend, the reader is referred to the Web version of this article.)

Using the dataset of all collection methods combined, PCA of exudate metabolic compounds showed clear separation by collection method along PC1 (60.9% of the variation explained, PERMANOVA main effect of collection method *F*_4, 60_ = 83.8, _p_*R*^2^ = 0.77, *p* < 0.001) and a species separation effect on PC2 (7.3% of the variation explained; PERMANOVA main effect of species *F*_2, 60_ = 8.8, _p_*R*^2^ = 0.04, *p* < 0.001 Supp. Fig. 3a), with a significant interaction between collection method and species (PERMANOVA collection method and species interaction *F*_8, 60_ = 2.6, _p_*R*^2^ = 0.05, *p* < 0.005). Due to distinct clustering on the PCA, leachate and hybrid data (normalised for root biomass) were analysed separately. Here, species-specific clustering was apparent across both datasets (Fig. 3) with collection method affecting metabolic profiles of the hybrid dataset (Fig. 3a). These effects were independent of the block design of the experiment, which showed no clustering (Supp. Fig. 3b and c). Both datasets contained similar classes of metabolites, but leachate had a higher proportion of alkanes and sugars, and a lower proportion of amino acids and unannotated metabolites (unknowns; Supp. Fig. 3). In the dataset without leachates (consisting of 761 aligned putative metabolites) there was also overall greater diversity of metabolites found, including secondary metabolites not detectable in the leachate. In this dataset, D and hybrid collection methods clearly separated along PC1 (61.2% PERMANOVA main effect of collection method *F*_3, 48_ = 39.8, _p_*R*^2^ = 0.47, *p* < 0.001) and separation between species became more apparent on PC 2 (10.8% of variation explained, PERMANOVA main effect of species *F*_2, 48_ = 38.7, _p_*R*^2^ = 0.30, *p* < 0.001) but there was no longer any interaction (PERMANOVA main effect of interaction *F*_6, 48_ = 1.5, _p_*R*^2^ = 0.05, *p* = 0.146). In the leachate only dataset (consisting of 135 aligned putative metabolites) *T. repens* clearly separated from the other species along PC1 (63.7% variation explained, PERMANOVA main effect of species *F*_2, 12_ = 10.8, _p_*R*^2^ = 0.64, *p* < 0.001; Fig. 3b).

Similar to PCA, *K*means clustering predominantly separated collection methods (Supp. Fig. 5). The four distinct clusters in the dendrogram separated mainly by collection method. Many of the detected metabolites had the highest representation in the damaged roots treatment (D; cluster 1, Supp. Fig. 5) and decreased across H0, H3 and H7 respectively (clusters 3–4). Additionally, the species displayed dissimilarities in their metabolite profiles irrespective of collection method (Supp. Fig 5). This was clearest in *T. repens,* which had a greater metabolite intensity than the other species and clustered together despite the effects of collection method (cluster 2). *R. acetosa* and *H. lanatus* were not separated, indicating that collection method had greater influence over the metabolome than species.

**Fig. 4:**
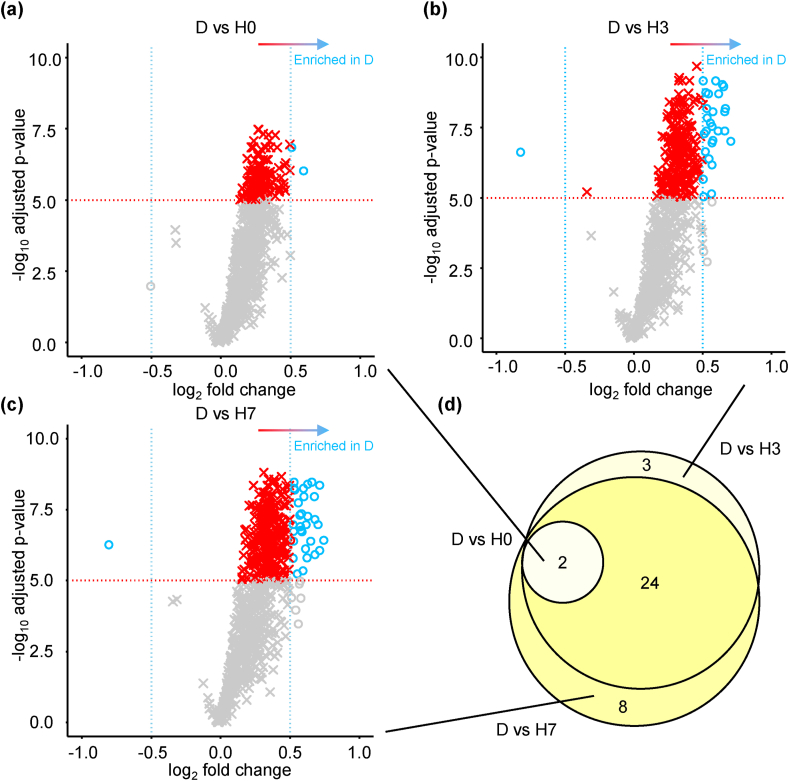
**Significant enrichment of metabolites in damaged root exudates compared to hybrid collection methods.** Volcano plots of adjusted *p*-values against fold change indicating the enrichment of metabolites in exudates from damaged roots compared to hybrid collection method with 0 days recovery (a), 3 days recovery (b) and 7 days recovery (c). Cut-offs for significant enrichment were selected at *p*-value (-log_10_) > 5 and fold-change (log_2_) > 0.5 or < −0.5. The overlap in the identities of these significantly enriched metabolites is indicated by venn diagram (d) and their putative identities are annotated in Table 1.

To test the extent to which exudates collected using hydroponic methods of increasing recovery period shared compounds with the extracts from damaged roots, we used volcano plots for binary comparisons of D *vs* H0, D *vs* H3, and D *vs* H7. Fewer metabolites (two) were significantly enriched in D compared to H0 (Fig. 4a) than in D compared to H3 (27; Fig. 4b) and compared to H7 (32; Fig. 4c), indicating that H0 was more similar to D than H3 and H7. While metabolites enriched in D compared to H3 and compared to H7 showed a large overlap, there were a number of metabolites specific to each comparison (three in D *vs* H3 and eight in D *vs* H7; Fig. 4d). The putative identities of metabolites enriched in D were determined, although this was not possible for 21 out of the 36 metabolites (58%) because they lacked a >80% match or were not annotated in the GOLM database (Table 1). Of those metabolites where identification was a close match, we found fatty acids (myristic acid), amino acids (isoleucine and malic acid) and sugars (arabinose and fucose). Interestingly, only one metabolite, with a 97.5% match to hydroxylamine, was significantly enriched in H3 and H7 compared to D (Table 1). Similar patterns were seen when the same comparisons were performed on data for individual species (Supp. Fig. 6 a–c), but species specificity in the exact metabolites enriched in damage was apparent (Supp. Fig. 6 d; metabolite identities are annotated in Supp. Data 1). Finally, to test the impact of hybrid recovery stages on exudate metabolome, volcano plots were again run comparing H0 *vs* H3 and H0 *vs* H7. This was performed on a dataset extracted and pre-processed without D samples to avoid bias during relativisation. PERMANOVA on this data indicated a significant effect of collection method (*F*_2, 36_ = 5.0, _p_*R*^2^ = 0.09, *p* = 0.004) and a significant effect of species (*F*_2, 36_ = 24.8, _p_*R*^2^ = 0.49, *p* < 0.001) but no significant interaction between collection method and species (*F*_4, 36_ = 1.2, _p_*R*^2^ = 0.05, *p* = 0.276). Metabolite enrichment was very low, and only a handful of significant metabolites were identified (including tyrosine enriched in H0 *vs* H3 and malic acid enriched in H0 *vs* H7; Supp. Fig. 7).

**Fig. 5:**
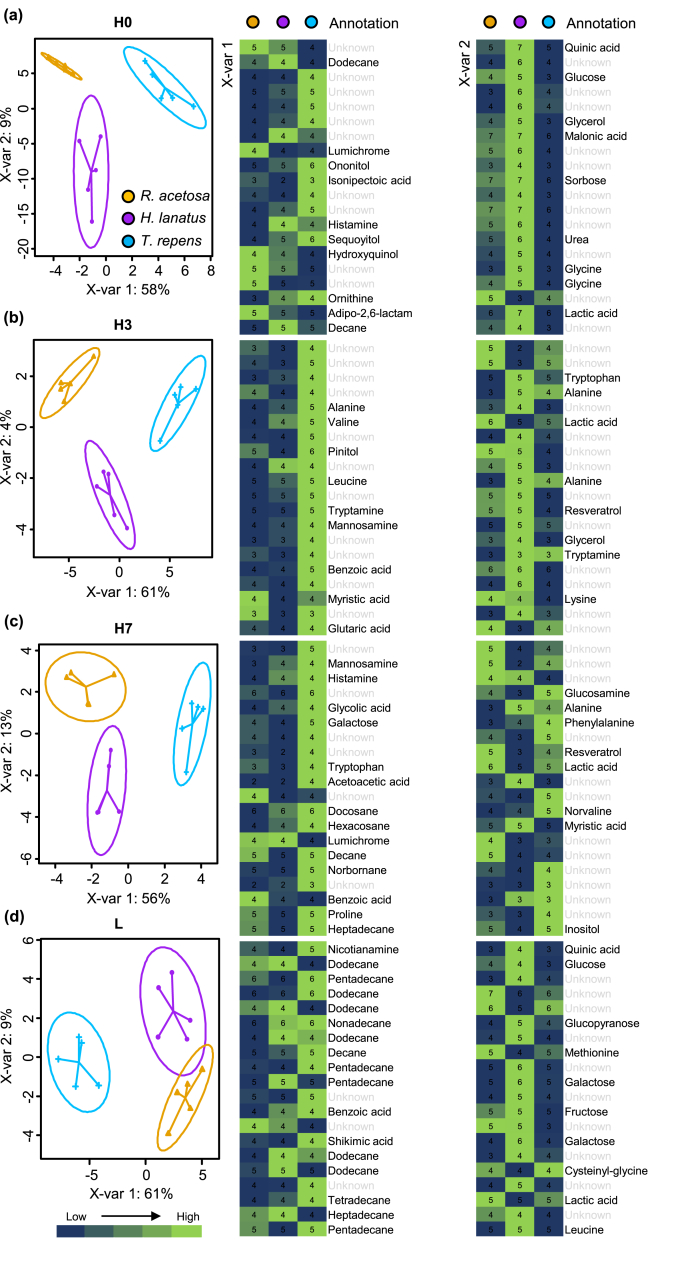
**sPLSDA of species-specific root exudate profiles across different collection methods.** Species differences within Damaged plants (a), 0 days recovery (b), 3 days recovery (c), 7 days recovery (d), and Leachate (e) treatments. The top 20 compounds that are most discriminatory between the species are presented in heatmaps to the right of the PLSDA plots, with colour indicating the relative average intensity for each species. Numbers within heatmap indicate log-values of metabolite intensity. Metabolite putative identities (>80% match in GOLM database) are annotated to the right of the heatmap for each axis. Compounds that could not be annotated, or were below the 80% threshold, are annotated ‘Unknown’ and shaded in grey. (For interpretation of the references to colour in this figure legend, the reader is referred to the Web version of this article.)

### 3.4 Species differences in exuded metabolites

To zoom in on the extent to which the different collection methods separated the root exudate metabolic profiles of the different species, we performed a series of supervised sparse partial least squares discriminatory analyses (sPLS-DA; Fig. 5). The model had good fit across each comparison (Balanced error rate <0.33, although we do note the relatively small number of samples used in these analyses) and it was evident that for each collection method two axes were sufficient to explain the variation in the data. In all cases the 3rd axis separated mostly intra-species variation and was therefore excluded from the model. Hence, the metabolites most important on axis 1 and 2 were putatively identified and annotated based on their GOLM database entries. The relative intensities of the top 20 of these metabolites in the different species were visualised in heatmaps with their putative identities (Fig. 5), although many we were unable to identify (35–55%).

Within each collection method, the separation between species on *X*-variable 1 was driven by metabolites that were enriched in the exudates of *T. repens*, with lower concentrations in exudates of *R. acetosa* or *H. lanatus* (Fig. 5). *X*-variable 2 contained a sizeable number of metabolites that were enriched in exudates of *H. lanatus* and some that were enriched in *R. acetosa*. Of the metabolites we could identify in H0, ononitol, isonipectoic acid, sequoyitol and ornithine were enriched in *T. repens* exudates, lumichrome, hydroxyquinol and adipo-2,6-lactam were enriched in *R. acetosa* exduates, and histamine and the hydrocarbons decane and dodecane were enriched in *H. lanatus* (Fig. 5a). *X*-variable 2 for H0 consisted of metabolites enriched in *H. lanatus* including glycine, quinic acid, malonic acid, lactic acid, glucose, sorbose, glycerol, and urea. In H3 and H7 there were only a few metabolites enriched in *R. acetosa* exudates (myristic acid, lumichrome and benzoic acid) on *X*-variable 1; all other metabolites were enriched in exudates of *T. repens*. These included amino acids (alanine, valine, leucine, histamine, tryptophan, proline and products involved in their biosynthesis/catabolism such as benzoic, glutaric and glycolic acid), diverse sugars (mannosamine, pinitol and galactose), hydrocarbons (decanes), the biocidal norbanane, the alkaloid tryptamine and metabolites involved in fatty acid metabolism (acetoacetic acid, myristic acid; Fig. 5b and c). On X-variable 2, *H. lanatus* exudates were enriched in certain amino acids (lysine, alanine, tryptophan), glycerol and the stilbene resveratrol. Lactic acid, resveratrol and lysine were enriched in exudates of *R. acetosa*.

In leachates, *X*-variable 1 consisted predominantly of hydrocarbon decanes present at higher levels in *T. repens*, along with benzoic acid, shikimic acid and nicotianamine. Some discriminatory metabolites were present in *X*-variable 2 and enriched in the *H. lanatus* rhizosphere, including the amino acid leucine, quinic acid and various sugars (glucose, fructose, galactose and glucopyranose; Fig. 5d). Methionine and lactic acid were enriched in *R. acetosa* leachates along with the dipeptide cysteinyl-glycine, which was also enriched in *T. repens.*

For a powerful indication of species-specific root exudates across the three species we applied volcano plots on a combined dataset of all three hybrid conditions (H0, H3 and H7). This analysis further highlighted metabolites that were specifically enriched in exudates from the three species (Supp. Fig. 8), with very few compounds enriched in *H. lanatus* against *R acetosa* (Supp. Fig. 8a) and the majority of compounds showing enrichment in *T. repens* against *H. lanatus* (Supp. Fig. 8b) and *R. acetosa* (Supp. Fig. 8c) with a lot of overlap between these metabolites (Supp. Fig. 8d). The identity of these metabolites are summarised in Supp. Table 2 and include amino acids, sugars, organic acids, cyclitols and purine, showing continuity with metabolites identified from the PLSDA.

## 4 Discussion

We set out to assess the suitability of a hybrid method for collecting root exudates compared to the more common method of leachate collection, and to evaluate the effect of a post root-wash recovery period on root exudate quantity and composition. We hypothesised that without recovery, the amount of C captured and the composition of the exudate would be more similar to extracts from damaged roots than after a 3- or 7-day recovery period. We also hypothesised that hybrid collection methods would provide a more identifiable and distinct metabolomic profile compared to leachates because of the absence of complex rhizosphere related compounds. In line with our hypotheses, we found that after recovery both metabolic profiles and C concentrations became more dissimilar to damaged root extracts. Although both hybrid collection methods and leachates demonstrated separation between species (Fig. 3), the important metabolites identified using the hybrid collection method were very different to leachate, illustrating the methods provide different information about the plant-soil system.

We found a clear impact of increasing root recovery period on root exudate quantity and quality. Specifically, a period of root recovery minimised the signal of root damage in the collected exudates. The amount of C in exudates collected immediately after washing (H0) was higher, while after 3 and 7 days of root recovery (H3 and H7) the amount of C decreased (Fig. 2), suggesting cell contents may by leaching out without a recovery period applied – although this varied between species. Exudate composition was similarly compromised without a period of root recovery: H0 profiles were compared to D (Fig. 3) revealing fewer significantly enriched (*i.e.* increased), damage associated metabolites as compared to either H3 or H7 (Fig. 4). Interestingly, the metabolites enriched in D were not specifically stress or damage associated *per se* (Table 1) but instead were probably representative of cell contents due to leaching of root internal metabolites from acute, and not sustained, cellular damage. For instance, damage associated metabolites such as jasmonates, like the hormone jasmonic acid (JA; Koo, 2018), or defence molecules such as alkaloids (Matsuura and Fett-Neto, 2015) were not found - this may be a limitation of the targeted method employed although, intriguingly, isoleucine, the amino acid conjugate of JA-isolecuine, was identified.

Recovery is further evidenced by the decreasing intensity of all the damage-associated metabolites with increasing recovery period (including myristic acid, glucopyranose, gluconic acid and arabinose; Table 1), and little to no enrichment of metabolites in H3 and H7 compared to damaged roots (only 1 metabolite, hydroxylamine; Fig. 4b and c). Previous studies that used the hybrid method (Aulakh et al., 2001; Canarini et al., 2016; Lucas García et al., 2001; Ström et al., 1994) placed washed roots immediately in the hydroponic collection medium, without any period of acclimation or recovery (Oburger and Jones, 2018). Our results indicate that, despite extra care been taken during root washing, this practice introduces significant bias from root damage (which may impact some species more than others; Supp. Fig. 6), both in terms of quantity and quality of root exudates, although impacts of different growth stages of the plant cannot be ruled out (Jones and Darrah, 1993; Oburger and Jones, 2018). Similar methods, such as the exudation trap on trees, do tend to apply a recovery period, and have been used to study both quantity of exuded C and metabolomics (Gargallo-Garriga et al., 2018; Phillips et al., 2008; Preece et al., 2018; Tückmantel et al., 2017). However, these methods collect exudates from a root section and not from a whole root system, and roots are typically left in hydroponic collection solution for longer periods (~24 h) after only very short periods in a recovery solution (~2 h), which could confound metabolomic investigation due to increased metabolite turnover and plant re-uptake (Vranova et al., 2013).

Leachates extracted a number of different classes of metabolites than the hybrid collection method (Supp. Fig 4; Fig. 5), and the size of the leachate dataset was smaller (135 compared to 761). This smaller size, and reduced diversity of metabolites, may result from lower sample numbers, but could also indicate that GC-MS is not the most discerning method for leachate analyses. Indeed, the C concentration of leachates was much higher than that of hybrid-collected exudates (Fig. 2). Many soil-associated metabolites, as well as dissolved organic carbon and humic substances, are likely to be collected in leachates (Pétriacq et al., 2017; van Dam and Bouwmeester, 2016), and LC-MS generates a diverse profile of secondary metabolites. Previous work has highlighted the speed of microbial degradation of root exudates, by demonstrating that accumulation of organic acids (acetate, glucose and citrate) does not occur in non-sterile rhizospheres of tomato seedlings (Kuijken et al., 2015). Although leachate collection methods are liable to contain an array of breakdown products, which obfuscates the origin of the identified metabolites, they have a number of advantages: they provide an integration of the effect of root exudates on the soil environment, and they allow for repetitive, non-destructive sampling of water-soluble metabolites. As the two methods (hybrid and leachate) measure different aspects of the rhizosphere chemical network (plant input and chemical context of the rhizosphere), the choice of which method is most appropriate depends on the study system used and the hypotheses being tested. A combined approach, as we have undertaken here, provides information on both the exuded metabolites and potentially on their chemical ‘fate’ in the rhizosphere, or at least the chemical context of the rhizosphere into which they have been exuded. In this study, both methods were appropriate to separate between species and provided us with information on the biological activities that these metabolites might have in the rhizosphere, but further exploration of leachate using alternative analytical methods, like LC-MS, may be useful (Swenson and Northen, 2019).

We found clear differences in root exudate quantity and quality between the three grassland species. In particular, *T. repens* had the highest exudation rate per unit of root biomass (Fig. 2) and the highest chemical diversity and abundance (Fig. 5c and d). While we found that exudate and leachate metabolite profiles of *H. lanatus* and *R.acetosa* were more similar to one another than to *T. repens* (Fig. 3), sPLS-DA allowed us to identify some metabolites enriched in these species (Fig. 5). As confirmed in the heatmap and subsequent volcano plot (Supp. Figs 5 & 7), many metabolites were enriched in *T. repens* exudates, denoting that exudation chemistry is particularly important for the rhizopshere biology of this species. Plant biomass allocation patterns indicated *H. lanatus* and *R. acetosa* had similar root:shoot ratios, but with distinct root trait profiles: *H. lanatus* had higher root volume, diameter and surface area and *R. acetosa* invested in higher root tissue density and root dry matter content (Fig. 1). *T. repens* by contrast had the lowest root biomass and highest root/shoot ratio, highest root N and SRL (Fig. 1). These patterns are consistent with earlier observations in *T. repens* and other members of the Fabaceae (Roumet et al., 2006) and are likely due to their strong association with N-fixing *Rhizobia*. The abundant presence of hydrocarbons we observed in the leachate of *T. repens* (Fig. 5) may demonstrate a pathway to recruit beneficial microbes, such as certain *Pseudomonads* that have the ability to metabolise certain alkanes (Cimmino et al., 2014; Molina et al., 2013). Discriminating the identity and role of these hydrocarbons in root exudates would benefit from techniques where hydrocarbon containing molecules, like terpenoids, are better retained and represented (such as reversed-phase liquid chromatography-MS, Narasimhan et al., 2003; Strehmel et al., 2014; Pétriacq et al., 2017).

Our results indicate that the hybrid approach is a suitable alternative to leachate collection, or pure hydroponic growth. While recovery may be necessary to ensure damage associated signals are restricted, limiting this time period can allow a relatively high-throughput assessment of species specific exudate profiles in different experimental systems (*e.g.* Williams et al., 2021) with an emphasis on obtaining ecologically valuable data. There is utility in this technique for broad spectrum approaches, such as liquid chromatography-MS, where different columns can be used accurately characterise a large range of metabolites of varying polarities. Alternatively, the hybrid technique could be adapted to provide spatial data of fine-root exudation processes using MS imaging techniques such as desorption electrospray ionization or matrix-assisted laser desorption ionization. These latter approaches may be more sensitive to damage impacts (as discussed previously; Pétriacq et al., 2017) and may therefore require longer recovery periods to be applicable. However, while the hybrid technique has certain advantages, it is not the *de facto* method of exudate collection as, with all collection techniques, there are unavoidable penalties to be considered. With hydroponics the system is artificial, with leachate the sample is marred by high levels of soil activity, with single root extraction obtaining enough exudate to analyse increases the risk of degradation, and with the hybrid collection method changing the system from soil to hydroponics, plus the root cleaning process, will likely have pleiotropic impacts on plant physiology. The choice of technique, therefore, must be context dependent and informed by the desired output.

Overall, we show that both hybrid exudate collection and leachates are useful tools for identifying exuded metabolites and rhizosphere chemicals at the species level. These can be indicative of their growth strategy, for instance allelochemical exudation, and may correlate with other well-known aspects of their biology, such as exploitative growth and associated root traits (Grime, 2006; Williams et al., 2021). Here we show that hybrid root exudate collection methods can yield ecologically relevant root exudates, and that a period of root recovery after washing may facilitate this. Without this root recovery period, exudates had much higher C concentrations, implying that root contents are leached and root-damage-associated metabolites present in the collected exudate sample may introduce inaccuracies when interpreting the function of root exudates. While leachates collected from the intact root-soil system are informative, the inability to discern whether metabolites originated from the plant, or microbial/soil processes is problematic when interpreting their functional relevance. Accounting for biases and limitations of root exudate collection methods is essential for correctly interpreting their role in ecosystems and their response to global change drivers. Our findings improve understanding of, and decision making in, the use of different root exudate collection methods.

## Author contributions

FTdV, RG and ALS conceived and designed the experiments; ALS, GF, HM, KH and HL performed the experiments and laboratory analyses; AW and FTdV analysed and interpreted the data with help from YX; AW wrote the manuscript with contributions from FTdV and the other authors.

## Declaration of competing interest

The authors declare that they have no known competing financial interests or personal relationships that could have appeared to influence the work reported in this paper.
